# Supplementary material for: Examining factors associated with mental health stigma and attitudes toward help-seeking among international college students during the COVID-19 pandemic
Source: PLOS Ment Health. 2024 Jun 20;1(1):e0000063. doi: 10.1371/journal.pmen.0000063 (PMC12798160; doi:10.1371/journal.pmen.0000063)
Supplement: Attachment — Submitted filename: Intl Student Help Seeking_Response to Reviewers.docx [file pmen.0000063.s001.docx]

**Response to Reviewers**

**Journal Requirements**

1. Please send a completed 'Competing Interests' statement, including any COIs declared by your co-authors. If you have no competing interests to declare, please state "The authors have declared that no competing interests exist".

We have revised the Competing Interests statement on the title page to state: “The authors have declared that no competing interests exist.”

2. Please amend your detailed Financial Disclosure statement. This is published with the article. It must therefore be completed in full sentences and contain the exact wording you wish to be published. If you did not receive any funding for this study, please simply state: “The authors received no specific funding for this work.”

We have revised the Financial Disclosure statement on the title page to read:  “The authors received no specific funding for this work.”

3. In the online submission form, you indicated that "Permission to share the full data set was not obtained from the participating institutions at the time of data collection. Requests for de-identified data may be made available upon request from the authors, or from the Duke University Center for Nursing Research, with an appropriate data transfer agreement."

 All PLOS journals now require all data underlying the findings described in their manuscript to be freely available to other researchers, either

1. In a public repository,

2. Within the manuscript itself, or

3. Uploaded as supplementary information.

This policy applies to all data except where public deposition would breach compliance with the protocol approved by your research ethics board. If your data cannot be made publicly available for ethical or legal reasons (e.g., public availability would compromise patient privacy), please explain your reasons by return email and your exemption request will be escalated to the editor for approval. Your exemption request will be handled independently and will not hold up the peer review process, but will need to be resolved should your manuscript be accepted for publication. One of the Editorial team will then be in touch if there are any issues.

We did not obtain permission from our institutional review, nor from the participating universities, to make the data obtained in this study freely available. I have responded by email to the Academic Editor to request an exemption from the public deposition requirement.

4. Please cite the tables within the main text/paragraph.

 	We now cite all three tables within the text.

**Comments to the Author**

Reviewer #1: Recommendation: Minor revision

This is a carefully conducted original study and a well-written paper which adds novel findings to the literature on mental health stigma and help-seeking. The findings have the potential to inform practice in relation to supporting international students in the U.S. and internationally. The findings are discussed appropriately in the context of the existing literature, and the authors are clear about what this study adds.

We thank the reviewer for their kind words and enthusiasm about the work.

My only major concern is that the limitations of the study – which are significant - are not highlighted in the discussion. The institutional participation rate and student response rate were low, and the overall sample size is correspondingly low, so the results may not be representative. Estimates have not been weighted to the national population of international students – do data exist to allow this? Could the exclusion of students not fluent in English introduced bias? Given the study's limitations, the findings should be interpreted with caution. For example on P 14 “…by showing that perceptions of interpersonal independence and interdependence MAY extend…”

We agree with the reviewer on this point and have added a new paragraph on page 15 acknowledging these limitations.

I would also like to query the definition of an ‘international student’ i.e. anyone not born in the USA. In a country like the USA with high immigration, surely a high proportion of university students were born elsewhere but would not be considered ‘international students’? The average time spent in the US suggests your survey did NOT pick up people who had spent most of their lives in the US, and I presume this is because the survey was distributed via International Student Services. Perhaps a clearer definition would be “students under the care of International Student Services or equivalent AND not born in the US” or similar?

We agree with the reviewer on this point and have revised our description of the definition accordingly on Page 5.

Reviewer #2: Thank you for the opportunity to review this well written manuscript.

Introduction:
Please make clear if Alharbi and Smith's work covers U.S. research.

We added the phrase “in the U.S. and other English-speaking countries” to this sentence on Page 3.

Please also define all acronyms before using them (US).

This adjustment has been made.

Can you adjust the sentence "Although these are valuable sources of support, these patterns of help-seeking can be problematic when they are relied upon instead of, rather than in addition to, professional support." At the moment, it appears to suggest that professional support should be used instead of family/ friend support, which may not be appropriate/ necessary in the first instance.

We replaced this sentence on Page 3 with “can be problematic if they are viewed as a replacement or proxy for needed professional support.”

"It is therefore critically important for studies to understand the influence of stigma and other key..." Add a comma after therefore, and also review the sentence as studies do not understand, people do.

We have revised this sentence on Page to read: “It is, therefore, critically important for researchers to conduct studies to understand the influence of stigma and other key…”

Method:
In reading that international students were defined as those not born in the US, I wonder what proportion would consider themselves to be international students. Some could have grown up in the U.S. or have significant familial connections here. While it appears the average student in this study has been in the country for only a few years, in an increasingly globalised world it is still likely that they may have connections in country.

As indicated in our response to Reviewer 1 and revised definition, only students who were recognized as an international student by International Student Services or the equivalent office at their institution were included in the study.

A statement around what level of statistical significance was used would also be beneficial.

We now note on Page 9: “P-values less than .05 were considered statistically significant.”

Similarly, explaining what % of surveys were excluded for being incomplete would strengthen this study, as would having a range for the response rate between the universities.

We have added the number of incomplete surveys and the range of response rates across the academic institutions on Page 6.

The method also lacks explanation for how the response rate was calculated - did the authors know the number of international students in each university?

We have now clarified on Page 6, “The average student response rate at 4 institutions who provided data on the number of international students invited was 4.6%”

When considering individualism vs. collectivism, was there any attempt made to identify cultures that are considered in the research to be more collective (rather than solely looking at the individual)?

Given the small sample size of the study, this type of subgroup analysis was not feasible.

Was data collected on the form of visa students had when studying e.g., refugee populations may face additional barriers than other international students.

We did not collect data on the form of visa students had. We now acknowledge this in the limitations paragraph on Page 15 and indicate that exploration of help-seeking differences based on immigration background is a valuable area for future study.

Results:
Given that both undergraduate and postgraduate students were surveyed, I find myself wondering if there were differences based on this, or indeed the course of study undertaken.

The variable undergraduate/postgraduate status was considered in preliminary models but ultimately omitted due to high inter-correlation with the “months in the U.S.” variable that was included in the analyses. We did not collect data on course of study, but agree this would be an interested question for future studies.

In Table 1, please change "Christian/Catholic" to Christian - it doesn't make sense to identify this denomination but not others. In addition, please separate atheist from none, as atheists believe that god does not exist (which in itself could be considered holding a stance more closely aligned with religious groups).

Religion was captured as an open-ended variable in response to the question “What is your religion?” We included the designation “Catholic” not based on any pre-conceptions of the researchers, but because of the large number of participants who listed it as a response to this item. Therefore, we believe it is appropriate to retain it in the demographic table. After careful consideration, we also respectfully disagree with the reviewer that atheism should be listed as a separate religion, as we feel justified in our choice to combine atheism with other non-religious groups.

In the paragraph before table 2, the authors write "Each 1-point increase in the level of prior contact with mental illness was associated with a .311 log count decrease in stigma in the univariable model (p<.001), and a .307 log count decrease in stigma in the multivariable model (p=.003)." This sentence should then lead on to a statement about the implication/ meaning of this.

Again, we respectfully disagree with the reviewer. This sentence in the Results section clearly describes the findings of the statistical test, which shows that prior contact with mental illness had a statistically significant, negative association with stigma. The implication/meaning of this finding is described in the Discussion section, which we believe is technically correct.

Discussion:
I would have liked a greater focus on potential study limitations. For example, acknowledgement of the low response rate. Were the universities who participated different in any way from those that did not? Did they have programmes to support international students? Perhaps it is my discipline, but I am inclined to ask for additional tangible recommendations on how to apply the research findings. At present, this is perhaps the main weakness of the study.

We now acknowledge the limitation of the low response rate and resulting threat to generalizability in the limitations paragraph on Page 15. The participating institutions largely reflected the broader characteristics of those that were invited to participate. We have also expanded our Discussion on page 13 to give a more detailed recommendation for how to apply the research findings: “Our novel findings provide a strong rationale to develop programs targeted specifically to reduce mental health stigma among international students, focused on bringing personal experiences with mental illness out of the shadows and into the campus discourse.”

Reviewer #3: This is an important area of study, which has not been adequately addressed in literature. However, the manuscript can be improved.

Introduction
The authors mention that “During the COVID-19 pandemic, international students faced a multitude of stressors, some of which were unique to this population; these included social isolation, campus closures in an unfamiliar country, travel restrictions, and challenges navigating an unfamiliar medical system. Even prior to the COVID-19 pandemic, acculturation and adjustment to the United States have been major factors influencing international student mental health”. Can the authors go on to describe how the mental health of international students have changed during the pandemic?

We added the following sentence and citations to describe how the mental health of international students has changed during the pandemic: “Preliminary data show that the pandemic had a considerable negative impact on international student mental health, including depression and anxiety, which was exacerbated by academic and financial stress.”^11,12^

Methods
The authors define an international student as “any student enrolled at the college or university who was not born in the U.S”. This is not in keeping with the widely accepted definition of an international student. The authors definition of international students can include students who were not born in USA, but have migrated to USA when they were very young, and are current citizens or permanent residents of USA. Can those who are not born in USA, but are current citizens/permanent residents of USA be labelled as international students? Were they excluded from the study? If so, this should be mentioned. If not, the authors should give justification why they were included as international students. The experience of those who migrated to USA when they were very young and entered USA collage/university after completing primary and secondary education in USA is very different from those who arrive in USA for tertiary education for the first time. Did the authors exclude those who have had primary/secondary education in USA? Can the authors please explain this in greater detail as it seems to be confusing.

As indicated in our response to Reviewers 1 and 2, as well as our revised definition, only students who were recognized as an international student by International Student Services or the equivalent office at their institution were included in the study.

Discussion
When giving reasons for not having any associations between demographic factors and stigma, the authors state “another factor may be historical: that the international students in our study who are men, are younger, or who are newer to the U.S. may now be less prone to stigma than they were in the past”. It is not clear what is meant by this.

We added the following phrase and citations on page 14 to clarify: “…may now be less prone to stigma than they were in the past due to global efforts in recent years to educate the public about mental health challenges, raise awareness, reduce stigma, and encourage mental health help-seeking.”^34,35^
