# Supplementary material for: Examining factors associated with mental health stigma and attitudes toward help-seeking among international college students during the COVID-19 pandemic
Source: PLOS Ment Health. 2024 Jun 20;1(1):e0000063. doi: 10.1371/journal.pmen.0000063 (PMC12798160; doi:10.1371/journal.pmen.0000063)
Supplement: Attachment — Submitted filename: Response to Reviewers_R2.docx [file pmen.0000063.s002.docx]

**Response to Reviewers**

Most of the reviewer comments have been addressed. Please make the following minor adjustments to ensure that all of these points have been covered well.

Thank you very much for your review and additional minor comments, which we have now addressed as outlined below.

The limitations of the study have been outlined in a section in the discussion. However, as you report, this is a relatively small study with relatively low response rates. These limitations mean the findings should be considered valuable and of interest but not definitive. The wording in other parts of the paper such the abstract, the statement of main findings (in the 2nd paragraph of the discussion) and your conclusion should reflect this. Please ensure you do not overstate the strength of the findings and implications in these places.

We agree on this point and have made minor revisions throughout the manuscript, as suggested, to acknowledge the small sample and exploratory nature of our findings. For example, we added the qualifier “in this sample” in the abstract, added the word “exploratory” and revised “diverse sample” to “small but diverse sample” on Page 5, and revised the second paragraph of the discussion as requested.

The decision you prefer re "Christian/Catholic" is not standard. Please reconsider this or add a short footnote to explain why you have done this at the bottom of the table.

We have now removed the word “Catholic” from the demographic table as originally requested.
